# Iceberg Scour and Shell Damage in the Antarctic Bivalve *Laternula elliptica*

**DOI:** 10.1371/journal.pone.0046341

**Published:** 2012-09-28

**Authors:** Elizabeth M. Harper, Melody S. Clark, Joseph I. Hoffman, Eva E. R. Philipp, Lloyd S. Peck, Simon A. Morley

**Affiliations:** 1 Department of Earth Sciences, University of Cambridge, Cambridge, United Kingdom; 2 British Antarctic Survey, Natural Environment Research Council, Cambridge, United Kingdom; 3 Department of Zoology, University of Cambridge, Cambridge, United Kingdom; 4 Institute of Clinical Molecular Biology, Christian-Albrechts University, Kiel, Germany; University of Plymouth, United Kingdom

## Abstract

We document differences in shell damage and shell thickness in a bivalve mollusc (*Laternula elliptica*) from seven sites around Antarctica with differing exposures to ice movement. These range from 60% of the sea bed impacted by ice per year (Hangar Cove, Antarctic Peninsula) to those protected by virtually permanent sea ice cover (McMurdo Sound). Patterns of shell damage consistent with blunt force trauma were observed in populations where ice scour frequently occurs; damage repair frequencies and the thickness of shells correlated positively with the frequency of iceberg scour at the different sites with the highest repair rates and thicker shells at Hangar Cove (74.2% of animals damaged) compared to the other less impacted sites (less than 10% at McMurdo Sound). Genetic analysis of population structure using Amplified Fragment Length Polymorphisms (AFLPs) revealed no genetic differences between the two sites showing the greatest difference in shell morphology and repair rates. Taken together, our results suggest that *L. elliptica* exhibits considerable phenotypic plasticity in response to geographic variation in physical disturbance.

## Introduction

Our understanding of how the environment impacts on species distributions and fitness is limited, and in many instances this issue is further complicated by undescribed genotype-environment interactions. The ability of organisms to respond to biotic and abiotic factors in their environment can result in the expression of highly variable phenotypes in the absence of genetic differentiation. This flexibility of response is often called phenotypic plasticity. Numerous examples of this have been described involving a range of behavioural, physiological, morphological and life history traits, which can significantly impact on fitness and survival, c.f. [1]. Very clear examples of phenotypic plasticity have been described from within the Mollusca, where shell shape and thickness vary with habitat e.g. [2]–[7].

Calcified shells provide protection from both predators and environmental agents, as well as support structures for organs and tissues and a means of sealing the soft tissue away from fluid loss or incursion of harmful substances. Although the shell is continually produced, it also requires active maintenance if damaged. Such damage may occur in a number of different ways, the incidence of which depends on habitat. The main method of damage for most species is biological (i.e. predators that crush the shell such as crabs, fish, gastropods and birds) [8], endoliths such as bacteria, polychaetes or clionid sponges [9]) or as a result of grazers, such as limpets that rasp surface algae [10]. There are also abiotic sources of damage, for example from moving rocks [11] or ice [12] or accidental breakage during burrowing [13]. Anthropogenic disturbance can impact on shells via collision with fishing or dredging gear [14] or by human trampling [15]. Finally chemical dissolution in either cold or acidic waters [16] may attack shells. If damage caused in these ways is not lethal, repair may be possible although at the cost of diverting energy from other functions, notably somatic growth or reproduction [17],[18]. However, it has been shown that some species activate a form of “preparative defence” by producing thicker shells in response to damage by predators e.g. [3] or environmental insults such as tidal emersion and ice impacts [5], [6]. These are easily measurable traits of phenotypic plasticity.

In this study we examine the ecological context of damage repair rates in the Antarctic anomalodesmatan bivalve *Laternula elliptica* (King and Broderip, 1831) and investigate its relationship to shell morphology. This large bivalve (exceeding 100 mm in length in very large specimens) is a keystone species of the Antarctic marine ecosystem [19], [20]. It is highly abundant with a circumpolar distribution and as the largest individual mollusc in the Southern Ocean with regard to live weight, it is one of the highest in terms of total ecological biomass [21]. As an infaunal filter-feeder, it plays a significant role in benthopelagic coupling [19],[22]. Hence in terms of its ecological importance, it has strong support for designation as an Antarctic model species [20]. This is enhanced by the extensive biological knowledge on this species [23] and its use in cross disciplinary research on ageing [24] and climate change [25]–[27]. Ocean acidification studies have demonstrated dissolution of shell with exposure to lowered sea water pH [26], [27]. As a consequence of potential climate change effects on this Antarctic model species, there are now increased efforts to understand shell manufacture and maintenance in this animal.

*Laternula elliptica* inhabits soft sediments with individuals burrowing to depths of more than 50 cm [21]. There are no crushing predators in the Southern Ocean [28] and their deep infaunal habit protects them from drilling trophonid gastropods, although it has been reported that the generalist notothenoid fish *Trematomus hansoni* feeds on them by cropping the siphons [29]. However, a major threat these animals face is iceberg scour. If they survive exhumation by icebergs, they lie on the sea-bed until they re-burrow but during this time damaged individuals may attract scavengers [30],[31]. Given that both shell repair and increased shell production can be costly [18], particularly in these cold waters and that such costs are predicted to increase under future climate scenarios [32], this study was undertaken to characterise the style of damage and repair sustained in a number of *L. elliptica* populations around Antarctica. The aim was to investigate differences in the incidence of damage repair between sites and also to compare the growth rates of damaged versus undamaged individuals. We also used Amplified Fragment Length Polymorphisms (AFLPs) to compare genetic structure between two populations of *L. elliptica* in which there are marked morphological differences.

## Materials and Methods

### Sample sites and animal collection

The Antarctic is not privately owned and collections were not made from any of the protected sites within Antarctica. The field studies did not involve endangered or protected species. Collections were made within Antarctic Act Permits numbers S7-06/2011 and S7-02/2010 as granted under sections 12 and 13 of the Antarctic Act 1994.

**Figure 1:**
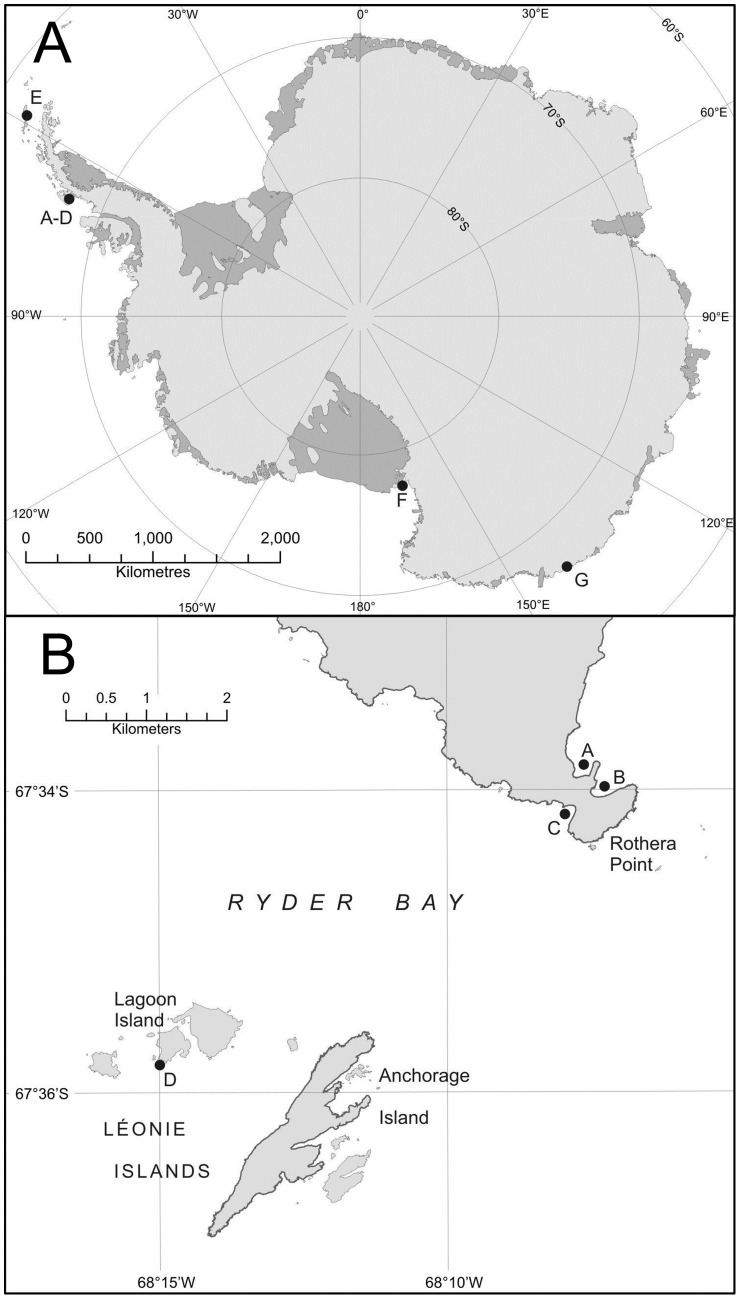
Locations of sampling sites. **A:** Sampling sites of *L. elliptica* from around Antarctica. **B:** Sampling sites of *L. elliptica* from around Adelaide Island. Abbreviations: A: North Cove, B: South Cove, C: Hangar Cove, D: Back Bay, Lagoon, E: Potter Cove, F: Ross Sea, G: Dumont D'Urville.

Samples of *Laternula elliptica* were collected by divers from 7 sites around Antarctica (Figure 1; Table 1) from depths of around 15–20 m. Sampling sites were chosen where there were differing exposures to ice movement (Table 1). Four of the sites (all in Ryder Bay, Antarctic Peninsula) are prone to iceberg disturbance. In a previous survey of ice impact at these sites 60% of the seabed in Hangar Cove suffered iceberg scour each year and this was 2.5× the scouring frequency of South Cove [33]. North Cove is adjacent to Hangar Cove and although not separately measured, the frequency of ice scour is similar and may potentially be slightly higher as the runway built between the two bays protects Hangar Cove and also Hangar Cove freezes over for longer periods, locking icebergs in place (L.S. Peck, pers obs.). Although considerable ice activity is known to occur at Back Bay, Lagoon Island, which is located further offshore in Ryder Bay, the relative frequency of ice scour is unknown. The remaining three sites are known to suffer less ice disturbance for various reasons. Winter Quarter's Bay, McMurdo Sound is protected because it is covered in sea ice which breaks out only very infrequently and for only brief periods (the last time was in 1998/1999 (Ed Butler, Antarctica New Zealand, pers. com.). The sea ice locks any icebergs present in place, and so the seabed is very rarely exposed to iceberg scour. Dumont D'Urville is similarly ice covered for much of the year, with an average ice free period of 3.9 months per year during the last 10 years (Météo France). The fine composition of the sediment in Potter Cove, King George Island and the reduced proximity of major tidewater glaciers (compared to Ryder Bay) suggest comparatively low iceberg impact, although icebergs have been seen to beach in the sampling area [31].

**Table 1:** Details of sampling sites, GPS co-ordinates and substratum type.

Site	Location	Co-ordinates	Substratum
Hangar Cove	Rothera Point, Adelaide Island, Ryder Bay	(67°34′S, 68°8′W)	Compacted cobbles and boulders with an overlying layer of silt of varying thickness (Brown et al. 2004; L.S. Peck pers obs..)
South Cove	Rothera Point, Adelaide Island, Ryder Bay	(67°34′S, 68°8′W)	Hard bedrock and areas of compacted cobbles with sediment of various grades between (Brown et al. 2004)
North Cove	Rothera Point, Adelaide Island, Ryder Bay	(67°34′S, 68°87′W)	Compacted cobbles overlain with layer of silt of varying thickness (S. A. Morley pers obs.)
Back Bay	Lagoon Island , Ryder Bay	(67°36′S, 68°14′W)	Consolidated pebbles and cobbles infilled with sediment (L.S. Peck, pers obs.)
Potter Cove	King George Island, South Shetland Islands	(62°14′S, 58°42′W)	Fine deep sediment (Philipp pers obs.)
Winter Quarter's Bay	McMurdo Sound, Ross Sea	(77°50′S, 166°39′E)	Fine deep sediment (S. A. Morley pers obs.)
Dumont D'Urville	Adélie Land, Dumont D'Urville Sea	(66°40′S, 140°00′E)	Fine deep sediment (J. Richard, pers obs.)

Conjoined valves were available from all samplings with the exception of those from Potter Cove, which consisted of dried, largely disarticulated individuals. In this case, data were collected for the left valves only to avoid problems of valve matching.

### Morphometric data

Valve lengths were measured (±0.1 mm) using digital vernier callipers. Shell thickness was measured for a sub-set of individuals from each site for which the outer shell layer was intact. Five measurements per specimen from each site were made, using a digital micrometer (with modified anvils, ±0.1 mm). Specimens were also aged by counting growth rings on the outer surface of the valve. These counts were made by a single operator (SAM) but also re-checked by another (LSP). Subsets of shells from each site were also aged by counting internal growth lines from sections cut through the chondrophore [34].

### Repair characterisation

For each site, all individuals were screened carefully, on both internal and external surfaces, for evidence of shell repair. Care was taken to distinguish between repaired damage and shells which were merely distorted by growing in close proximity to large clasts within the sediment. Particular attention was given to the position of the damage with respect to the valve margins, whether it affected both valves and whether shell material had been removed or just displaced. Where there was evidence of repaired damage involving cracking of the calcareous part of the shell the degree was scored as ‘low’, ‘moderate’ or ‘severe’ (see Table 2). Scores were recorded by a single operator (EMH) to ensure consistency.

**Table 2:** Criteria to score the level of shell damage and repair.

Damage Level	Characteristics of repair
None	No visible shell repair or damage
Low	Small areas of ‘hairline’ cracks or dents with little or no displacement of valve on either side (e.g. Figure 2A)
Moderate	Long (>10 mm) cracks about which evident displacement and re-attachment of shell fragments (e.g. Figure 3C & D)
Severe	Large areas of displaced and re-attached shell fragments affecting much of the valve. Sometimes damage affecting the hinge region, with repairs evident in either the chondrophore or buttress (or both) (e.g. figure 3A & B)

### Collection of genetic data

**Figure 2:**
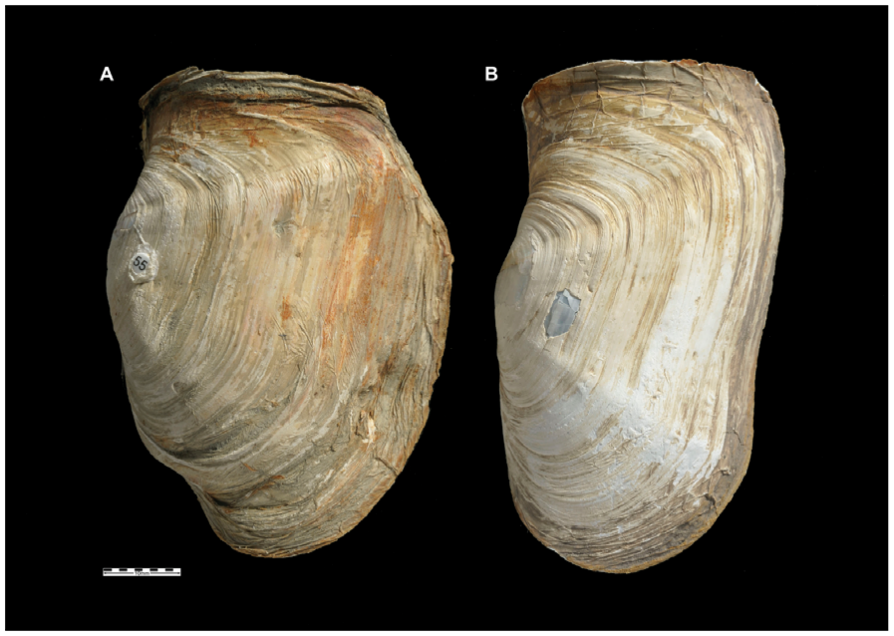
Photographs of typical undamaged shells. A: Hangar Cove; B: Potter Cove. Scale bar = 10 mm.

Gross morphological differences in shell shape were observed among many of the populations. For the two populations that differed the most in this respect (Potter Cove and Hangar Cove, Figure 2), in-house collections of tissue samples (96 individuals at each site) were available for genetic analysis using AFLPs. For each sample, total genomic DNA was extracted from a small piece of foot tissue using the Qiagen DNeasy tissue extraction kit following the manufacturer's recommended protocols. The AFLP protocol was the same as that used by Hoffman et al [35] , with six different selective primer combinations being used (Table 3). PCR products were resolved by electrophoresis on standard 6% polyacrylamide sequencing gels and detected by autoradiography. Exposed X-ray films were assessed and if required, a second exposure was made for an adjusted time period. Gels were scored by eye and genotypes were entered manually into a Microsoft Excel spreadsheet.

**Table 3:** Primer combinations used for the AFLP selective amplification and numbers of AFLP polymorphisms generated for 192 individuals of *Laternula elliptica* (96 from each site).

*Taq*I primer (5′-3′)	*Eco*RI primer (5′-3′)	Number of polymorphic loci	Number of monomorphic loci	Polymorphism (%)
GATGAGTCCTGACCGACAC	GACTGCGTACCAATTCAGC	11	4	73.3
GATGAGTCCTGACCGACAG	GACTGCGTACCAATTCAGC	8	3	72.7
GATGAGTCCTGACCGACCA	GACTGCGTACCAATTCACA	15	5	75.0
GATGAGTCCTGACCGACGA	GACTGCGTACCAATTCAGC	11	1	91. 7
GATGAGTCCTGACCGACTG	GACTGCGTACCAATTCAGC	19	7	73.1
GATGAGTCCTGACCGACTG	GACTGCGTACCAATTCATG	4	2	66.7
**Total**		68	22	75.6

### Analysis of genetic data

The program Aflp-Surv v1.0 [36] was first used to calculate pairwise *F*_st_ values following [37] and to conduct a permutation test for overall genetic differentiation using 1000 permutations of the dataset. We then used the approach of Holsinger *et al*. [38] as implemented in the program Hickory v1.1 to estimate Bayesian analogues of *F*_st_ and *F*_is_, designated *θ* ^II^ and *f* respectively. This software allows the estimation of four different models. The first is a full model in which *θ* ^II^ and *f* are estimated simultaneously. Two alternative models assume that *f* = 0 (no inbreeding within populations) and *θ* ^II = ^0 (no differentiation among populations) respectively. Finally, because estimates of *f* can be unreliable for dominant datasets, the program allows the estimation of a final model in which *f* is free to vary. All four models were run using a burn-in of 50,000 iterations followed by a further 250,000 iterations, with values being retained at every 50^th^ iteration (as recommended 37]). Posterior distributions and trace plots were carefully scrutinized to ensure convergence of the Markov chain Monte Carlo sampler. Models were then compared using the deviance information criterion (DIC) which is analogous to Akaike's Information Criterion (AIC) and combines a measure of model fit (Dbar) with one of model complexity (*p*D, the effective number of parameters). Models with smaller DIC values are preferred, but a difference of more than 6 units is required to indicate strong support for one model over another [38].

### Outlier detection

To test for evidence of AFLP loci subject to selection, we used a recently developed Bayesian approach implemented within the program BayeScan [39] This approach generalises the method of Beaumont and Balding (2004) [40] to allow direct estimation of the posterior probability that a given locus is under selection. To summarise briefly, it is based on a logistic regression model of *F*_st_ that incorporates both locus and population as predictor variables, the latter accounting for variation both in the demographic histories of individual populations and in the magnitude of genetic drift among them. For each locus, the probability of being under selection is then inferred using the Bayes factor, which is calculated as the ratio of the posterior probabilities of two models, one that includes the locus and another that excludes it. These posterior probabilities are estimated using Reversible Jump Markov Chain Monte Carlo (RJMCMC). Based on Jeffrey's (1961) [41] scale of evidence, a Bayes factor between 3 and 10 is interpreted as ‘substantial evidence’ for one model being supported over the other. For our analysis, the estimation of model parameters was automatically tuned on the basis of ten pilot runs of 5000 iterations each. After an additional burn-in of 50,000 iterations, we then used a sample size of 10,000 iterations and a thinning interval to 50 as suggested by Foll and Gaggiotti (2008) [39], resulting in a total chain length of 550,000 iterations.

## Results

### Damage and repair

**Figure 3:**
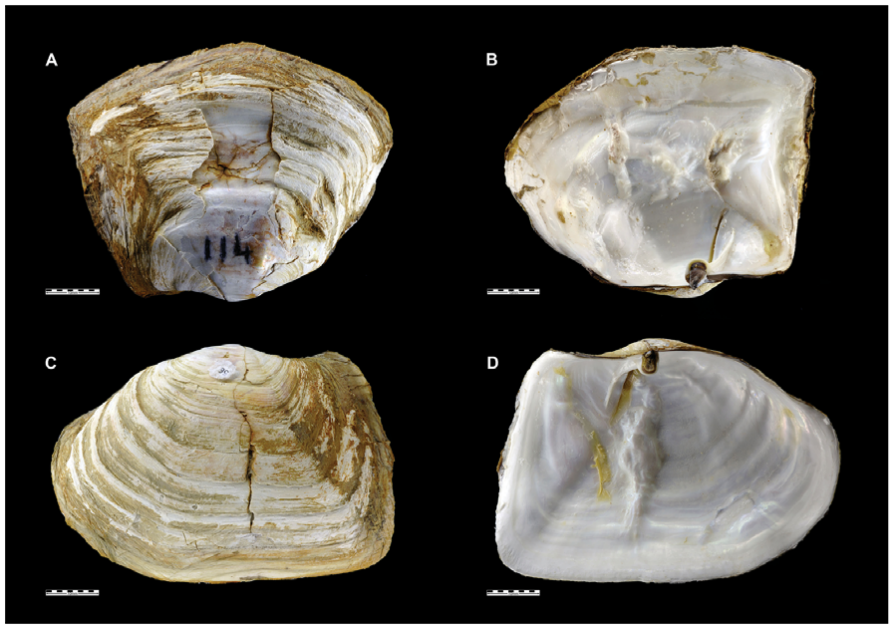
Photographs showing different styles of shell damage and repair. External damage (A;C) and associated internal repair (B;D) of shells from two individuals from Hangar Cove. Scale bar = 10 mm.

**Figure 4:**
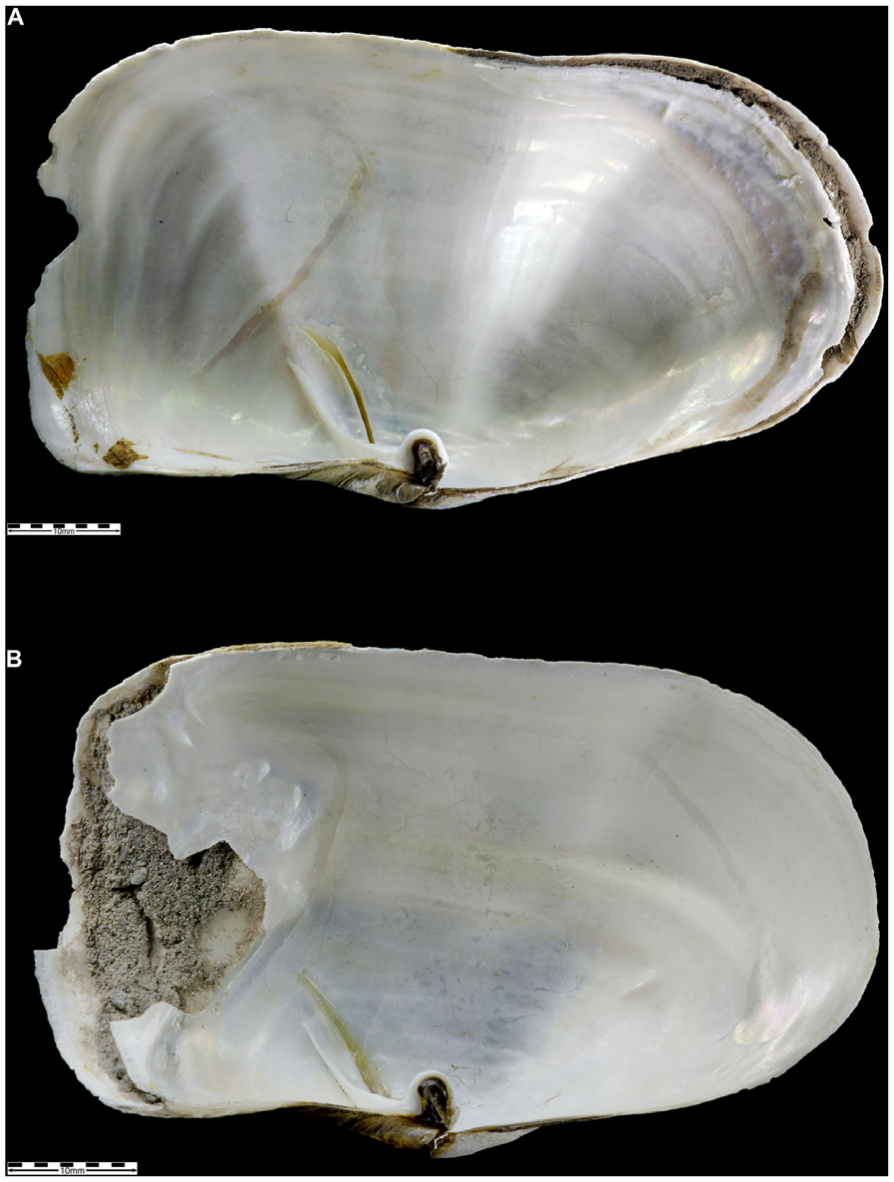
Photographs showing sediment incursion damage. Sediment deposits within the shell from two individuals from Potter Cove. Scale bar = 10 mm.

The incidence of shells with repaired damage varied markedly between sites and ranged from 1.7% to 74.2% (Table 4). The most prevalent form of damage, recorded most often in the Hangar Cove population (74.2% of individuals with repairs), involved distinctive cracking, producing jagged fragments, visible chiefly on the external surface (Figure 3 A; C). This damage was not associated with prominent growth checks and was notably asymmetric between the two valves. There was no sign of removal of shell material, rather that individual jagged fragments were displaced and then re-incorporated by subsequent shell deposition, sometimes at haphazard angles. In most cases the damage radiated from a particular point but in others the cracking was more radial. In some instances a considerable amount of organic material had been laid down during repair. The internal surfaces of these damaged shells were less disfigured, cracks being smoothed over by subsequent valve deposition (Figure 3 B; D). A particular feature of this style of damage was that in the vast majority of cases it could be ascribed to a single event and, therefore, the differences between ‘low’ and ‘severe’ damage could not be to the accumulation of multiple damages in the latter. The second type of damage was recognised where normal shell deposition had been interrupted, although with no associated shell damage, and the shell incorporated a layer of unconsolidated fine sand within the valve at either the posterior or the anterior end (Figure 4). This style of damage was restricted to the Potter Cove and Ross Sea sites.

**Table 4:** Morphometrics and levels of repaired damage in different populations.

Locality	N	Length range (mm)	Shell thickness (mm)	% repaired	Age range (years)	Damage score (N)	
Hangar Cove	67	30–98.5 Mean 62.0	0.859	74.2	0–17 Mean 9.5	None	16
						Low	19
						Mod	18
						Severe	14
North Cove	48	32.4–70.1 Mean 48.4	0.795	37.5	6–11 Mean 7.8	None	30
						Low	14
						Mod	4
						Severe	0
South Cove	45	39.8–95.0 Mean 63.2	0.622	42.2	4–12 Mean 7.8	None	26
						Low	11
						Mod	7
						Severe	1
Back Bay, Lagoon	29	44.4–87.4 Mean 65.6	0.671	41.4	7–14 Mean 10.0	None	17
						Low	5
						Mod	5
						Severe	2
Potter Cove	54	39.7–85.5 Mean 65.9	0.439	7.4	6–12 Mean 9.2	None*	50
						Low	2
						Mod	1
						Severe	1
Winter Quarter's Bay, Ross Sea	58	35.2–92.6 Mean 64.9	0.412	1.7	8–19 Mean 13.7	None*	57
						Low	1
						Mod	0
						Severe	0
Dumont D'Urville	21	46.2–74.3 Mean 63.5	0.502	28.6	7–14 Mean 10.5	None*	15
						Low	6
						Mod	0
						Severe	0

**Figure 5:**
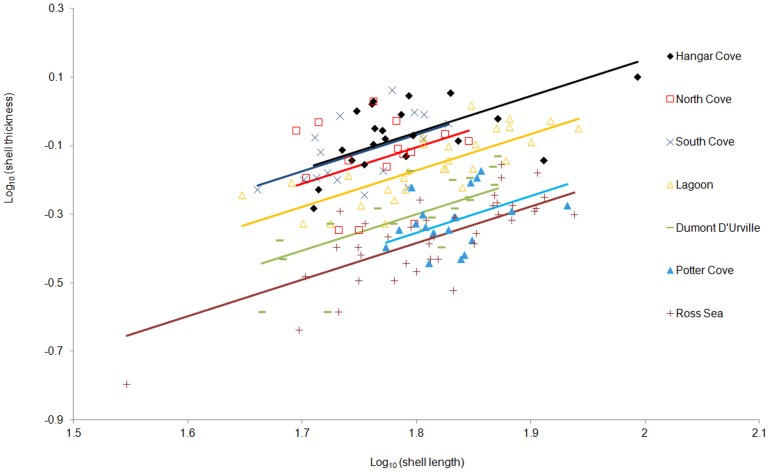
Relationship of shell thickness to shell length at each location. Slopes were not significantly different (F_6,166 = _1.0, p = 0.43), intercepts were (F_6,166 = _63.1, p<0.01), so the ANCOVA was recalculated with a common slope. Hangar Cove, log_10_(thickness) = 1.07log_10_(length)-1.98; North Cove, log_10_(thickness) = 1.07log_10_(length)-2.02; South Cove, log_10_(thickness) = 1.07log_10_(length)-1.99; Lagoon, log_10_(thickness) = 1.07log_10_(length)-2.09; Dumont D'Urville, log_10_(thickness) = 1.07log_10_(length)-2.22; Potter Cove, log_10_(thickness) = 1.07log_10_(length)-2.27; Ross Sea, log_10_(thickness) = 1.07log_10_(length)-2.30.

* denotes sediment incursions. Thickness calculations are derived from regressions in Figure 5 and solved for an average sized animal (63.1 mm shell length).

### Morphometrics

**Figure 6:**
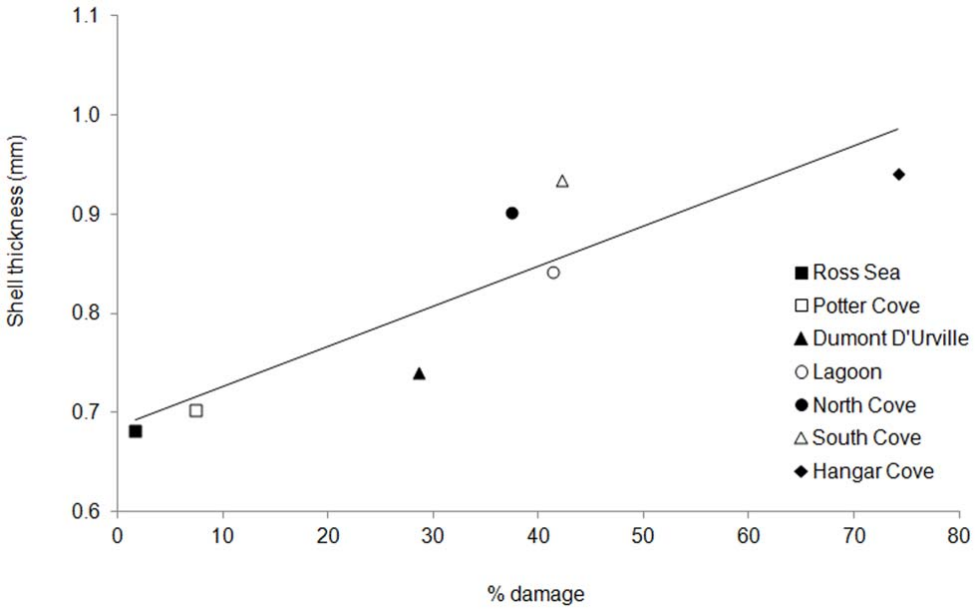
Regression between thickness and percentage damage of *L. elliptica* shells across the different sites. Calculated for the average sized animal (63.1 mm shell length). thickness = 0.0040.%damage+0.69 (F_1,6_ = 17.8, R^2^ = 0.78, p<0.01).

Morphological characterisation centred on the shell length, thickness and age structure of each of the populations. At each location, shell length and thickness were positively correlated (Figure 5). When shell thickness and length were log_10_ transformed residuals of ANCOVA analysis were normally distributed (AD = 0.44, p = 0.30) with a homogenous variance (Levene's test = 1.02, p = 0.41). Slopes of these relationships were not significantly different between sites (F_6,166 = _1.0, p = 0.43). When data were analysed with a common slope, elevations and hence shell thickness were significantly different between sites (F_6,166 = _63.1, p<0.01). Posthoc Tukey tests showed that shell thickness varied on a continuum from the thinnest (Ross Sea) population to the thickest (Hangar Cove) population. When shell thickness was calculated for the average sized animal (63.1 mm length) there was a significant regression (F_1,6_ = 17.8, R^2^ = 0.78, p<0.01) between % damage and shell thickness; thickness = 0.0040.%damage+0.69 (Figure 6). The effect of damage on thickness within a site was measured using shells from three sites (Lagoon, North Cove and South Cove), which all showed similar levels of damage (40%) and age ranges. The thickness of the shells was not significantly different whether they were damaged or not (nested ANOVA; F_3,59_ = 0.51, p = 0.68).

**Figure 7:**
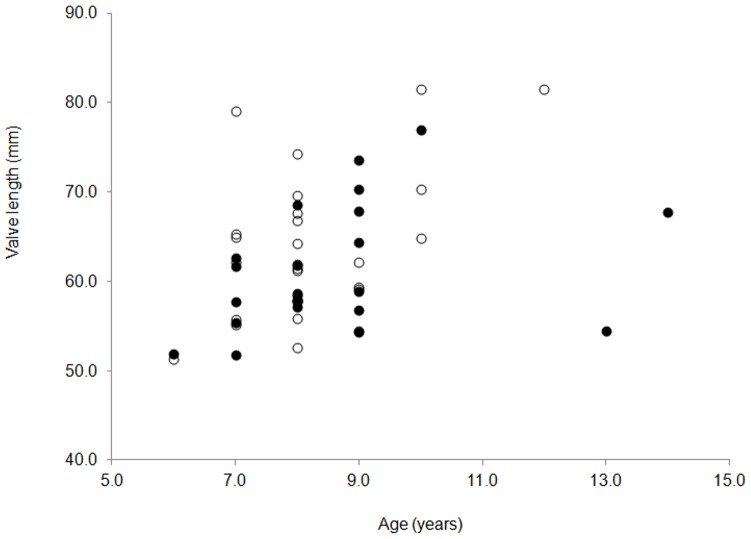
Comparison of the impact of damage and repair on growth rate in *Laternula elliptica* from Hangar Cove. Hangar Cove data split into two subsets (those showing no or low levels of damage (open circles) verses those with moderate or severe damage (closed circles) plotted against their relative ages. NB. Data sets range from 52–84 mm (length) and 6–14 years (age).

Ages derived from external growth ring counts (13.6±0.4, mean±sem) were not significantly different from the subset derived from internal bands within the chondrophore (13.8±0.4; T = 0.3, p = 0.78, n = 59). The maximum age in sampled animals ranged from 12–19 years across sites (Table 3). In order to investigate whether shell damage and repair influenced growth rate, the Hangar Cove data were split into two subsets (those showing no or low levels of damage verses those with moderate or severe damage). A plot of these data against their growth ring ages revealed no significant difference between the growth rate of the two groups (Figure 7) (ANCOVA; F_1,49 = _1.3, p = 0.25). Residuals from this analysis were normally distributed (AD = 0.67, p = 0.07) and of equal variance (Levene's test = 0.24, p = 0.63). This confirms the previous data on thickness and damage frequency, suggesting that damage had no discernible effect on the growth rates of the animals studied.

### Population genetic differentiation

Ninety six individuals each from Hangar Cove and Potter Cove were genotyped using six different selective primer combinations, yielding 90 AFLP loci, of which 68 (75.6%) were polymorphic (Table 5). A permutation test using 1000 permutations of the dataset found no evidence for genetic differentiation between the two populations (*F*_st_ = 0.0030, 95% CI = −0.0036–0.0081, *P = *0.83). Table 5 shows *f* and *θ* ^II^ values obtained from four different models of population structure using the program Hickory [38]. Although the full model yielded the lowest Dbar and DIC values, differences in DIC between the full, *f* = 0 and *θ* ^II^ = 0 models were small (i.e. less than 6 units), giving little reason to prefer any one of the models over the others [38]. Reassuringly, both the full and *f* = 0 models yielded *θ* ^II^ estimates that were very similar to the observed *F*_st_ value at 0.0038 and 0.0033 respectively. This implies good concordance between the traditional (*F*_st_) and Bayesian (*θ* ^II^) estimators of genetic differentiation employed. We also used the Bayesian approach to test for outlier loci in our AFLP dataset (see methods for details). However, none of the loci were identified as being influenced by selection.

**Table 5:** Bayesian analysis of *Laternula* population structure using the program Hickory (Holsinger *et al*. 2002).

Model	*f*	*θ* ^II^	95% credibility interval for *θ* ^II^	Dbar	pD	DIC
full	0.4928	0.0038	3.21×10^−4^–0.0094	671.94	75.68	747.62
*f* = 0	–	0.0033	3.28×10^−4^–0.0080	669.55	82.80	752.35
*θ* ^II^ = 0	0.4827	–	–	691.93	59.64	751.58
free *f*	0.4980	7	0.0305–0.1844	777.67	214.84	992.50

Estimates of *f*, *θ* ^II^ and the 95% credibility interval for *θ* ^II^ (see methods for details) are shown for each of four different models tested. Dbar is a measure of how well a model fits the data (models with smaller values are preferred) and *p*D indicates the effective number of parameters being estimated. The Deviant Information Criterion (DIC) is a model-choice criterion that takes into account both of the above. Models with smaller DIC values are preferred, but a difference of more than 6 units is required to indicate strong support for one model over another.

## Discussion

Repaired shell damage was recognised in *L. elliptica* from a range of Antarctic sites. This varied in frequency between values of 74.2% at Hangar Cove (Adelaide Island) and less than 10% at the Potter Cover and Ross Sea sites. There were two main types of damage present and these are sufficiently distinctive to allow an assessment of their causes. The lesser of the two styles of damage, characterised by the incorporation of a sediment layer within the shell (Figure 4) with no appreciable damage to the shell edge, was recognised at only the Potter Cove and Ross Sea sites where it was the dominant type. It is equivalent to that described by as ‘double walls’ in deep burrowing bivalves (e.g. *Lutraria* and *Panopea*) [13] and similar to that described in the razor clam *Tagelus plebeius* where it was associated with external marginal damage attributed to injuries caused by avian predators [42]. Presumably in each of these cases, the sediment layers resulted from some trauma whereby the periostracum was breached and sedimentary particles entered the extra-pallial space. It was noted that the Potter Cove and the Ross Sea sites have much finer sediment (Table 4), with higher sediment loading in the water column in Potter Cove [31] due to glacier melt water and perhaps storm activity. There is also evidence that because of this, the Potter Cove animals take in considerable amounts of sediment over the gills, which they then cover with mucus and excrete through pumping movements [31].

The dominant damage repair involved distinctive crackingthat produced jagged shell fragments, which were often displaced and then re-incorporated by subsequent shell deposition (Figure 3) and which frequently affected only one valve. Since *Laternula elliptica* is a deep-burrowing bivalve, living at depth that may exceed 50 cm below sediment-water interface [21], there are no obvious causes of such crushing damage while the individuals are in life position. Although it is known that deep burrowers may sustain repairable damage at the valve margins by closing their valves onto unyielding particles within the sediment this is not thought to be responsible for the majority of the damage described herein because such repairs are predominantly of arcuate chips removed from the anterior margin, afflicting both valves simultaneously [13]. We consider that the damage we have described must have been sustained either during or after exhumation of individuals from the safety of their burrows.

In most marine environments shell damage is commonly associated with the activities of durophagous predators but we reject this explanation for the crushing damage observed in *L. elliptica*. The Antarctic fauna lacks major durophagous taxa (i.e. no crabs, lobsters or sharks) [43] which might be capable of such causing shell damage. Additionally the style of damage to *L. elliptica* described herein is not consistent with the criteria established for the recognition of that inflicted by durophagous predators [44] because, in particular, it was not marginal, did not result in removal of shell material and was often present on one valve only. Most of the damage we report involves shell breakage distant from the valve to edge which appears to be the result of blunt force trauma, consistent with the brittle shell being pushed into the soft tissue inside without significant removal of shell material. The most obvious cause of this damage is exhumation by and collision with moving ice. Indeed, scouring action has been observed to exhume individuals of *L. elliptica* from within soft sediments [30], [31], [45] during which events some individuals are damaged [30]. Similar ice damage has been reported in Antarctic limpets [12]. We reject other theoretical abiotic causes. Although human disturbance, such as trawling and dredging, may exhume and damage infauna in this way [14], such anthropogenic causes cannot be responsible in Antarctica. In the mid latitudes, and in shallow water, infauna may be scoured out by storm activity and damaged by moving clasts [11], however, our animals were living at depths of 15–20 m which is below the level of wave disturbance. Furthermore wave disturbance is dampened by the presence of sea ice [46]. The argument that the damage was caused by ice impact is supported by the fact that the frequency and severity of damage was higher at sites with a higher frequency of iceberg scouring and absent at sites under near permanent ice cover where ice movement is negligible for most of the year. Substratum may also play a significant role, as the Potter Cove and Ross Sea samples have a different type of damage compared to the other populations. The Rothera populations generally live in a very rocky environment with varying levels of sediment in the different locations. Hangar Cove has patches of deeper sediment, compared to South Cove, but in the latter (and also the other Ryder Bay sites) there are larger sediment aggregates and more large boulders which could potentially provide a measure of protection from ice scour (L.S. Peck, pers obs.).

Ice scour is a major force in shaping the ecological characteristics of both the Antarctic [33], and Arctic [47] benthos. In Antarctica its effects take the place of durophagous predators in structuring communities that form in the shallow subtidal [43]. Although necessarily restricted to polar and sub-polar latitudes, ice scour nonetheless affects an estimated one third of the global coastlines [33] and in recent geological time will have been more so during glacial maxima, when northern hemisphere ice bergs repeatedly penetrated south to latitudes as low as 41°N [48]. Although global climate change may ultimately remove the threat of ice damage to marine communities, evidence suggests an immediate effect of warming will be the release of more ice-bergs and thus more scouring [49].

Shell damage caused ice impacts may be immediately fatal, although there are no data available to assess mortality rates [31]. Once exhumed, individuals are at risk of further ice impact and, particularly if damaged, leaking body fluids will attract scavengers and predators, such as the asteroid *Odontaster validus* and the nemertean worm *Parborlasia corrugatus* [30] until they have re-burrowed. The integrity of the shell is also important in this species for burrowing [45]. Especially large *L. elliptica* individuals might be more prone to predation and vulnerable to iceberg scour as they were found to require longer times for reburial [31], [50] and showed higher mortality rates after injury compared to smaller individuals [31]. The ability to repair valves damaged by iceberg scour is, therefore, an important mechanism for survival and population structure in this species.

Consistent with the data on shell repair frequencies, we also find a positive correlation between shell thicknesses and physical disturbance (Figure 6; Table 4). Interestingly, the genetic survey using AFLPs, which are sensitive to even very minor genetic differences [51], revealed no significant differences between the thickest shelled population at Hangar Cove and those of the thinnest shells from the geographically most distant and relatively undisturbed site at Potter Cove (Table 5). This is reminiscent of the Antarctic limpet *Nacella concinna* in which a continuous cline in shell shape and thickness has also been reported within a genetically homogeneous population, but in this example, associated with depth at one site [6]. The thickest limpet shells were found in the inter-tidal region, which is impacted by brash ice and ice bergs with a high frequency in the summer. The shells become increasingly thinner with depth, which correlated with frequency of ice berg impact. A lack of population structure in *L. elliptica* is consistent with the cosmopolitan distribution of this species around Antarctica and its mode of reproduction [52]. In this respect, the situation is what would be expected of broadcast spawners with long planktotrophic larval stages [6],[53] [54].

The lack of genetic differentiation at a genome-wide suite of AFLP markers, coupled with the marked relationship between thicker shells and more disturbed environments, strongly suggests that morphological differences among sites are due to phenotypic plasticity. As individuals in disturbed environments produce thicker shells, an obvious test of this might be to perform a common garden experiment. However organising the simultaneous collection and subsequent and movement of animals between Antarctic bases almost 3,000 km apart is not only logistically difficult, but also contravenes the Antarctic treaty, Resolution 6 (2011) ATCM XXXIV – CEP XIV, Buenos Aires prohibits the introduction of non-native species into the Antarctic area and also included movement of species between locations within the region. Our findings are consistent with a number of studies of marine and freshwater molluscan species that have reported marked phenotypic differences in the absence of population genetic structure (e.g. [6] [7]). However, although AFLPs allow us to rule out a link between shell morphology and genome-wide differentiation, we cannot exclude the possibility that one or a small number of genes could be implicated. This is not supported by our data, with our outlier analysis finding no evidence for any of the AFLP loci behaving non-neutrally, as might be the case if they were linked to genes subject to divergent selection pressures at the two sites. However, the power to detect such associations depends on numerous factors including the number of markers deployed, the number of genes involved, and the rate at which linkage disequilibrium decays with physical distance along chromosomes. Consequently, a better approach might be to target genetic polymorphisms within specific ‘candidate’ genes associated with shell deposition. However, this is not yet feasible for Antarctic species because genomic data are usually lacking. In a recent effort to improve our knowledge of the genomics of *L. elliptica*, we developed a comprehensive transcriptome database for *L.elliptica* comprising 18,290 putative transcripts [55]. In spite of the large number of transcripts only 17% of these could be annotated, due to the lack of a closely related model species with significant functional gene annotation. In that study, the closest comparator species were the blue mussel, *Mytilus edulis* and the gastropod snail, *Lottia gigantea*, both of which were largely unannotated bar assignment of a clone ID. It is unlikely that any one of these is responsible for shell thickness in this mollusc and its is more likely to be a product of either a single, or potentially several, quantitative trait loci involving many genes, some of which will have no annotation in the databases. Consequently, a targeted gene approach in this species will be non-trivial and have to await further genomic and bioinformatic advances.

Phenotypic plasticity in shell morphology across geographic regions has been described in other molluscs such as *Littorina* [2], mytiloids [3] and *Nucella* [56] and has often been correlated with environmental factors. These include wave exposure [2], temperature [57] and predation [3], [58]. Perhaps surprisingly in this study, repaired shells were not thicker than those that had not been damaged, suggesting that an indirect mechanism must be responsible for the correlation between shell thickness and frequency of damage. Given the data and the correlation of damage with disturbance, the question arises as to the environmental cue(s) which could produce this. It is possible that a combination of disturbance and chemical cues are involved. Mechanical disturbance does affect phenotype (reviewed in [58]) and has been correlated with shell thickness in another Antarctic species, *Nacella concinna* [6]. Experiments have been performed on temperate molluscs using chemical extracts or physical exposure of predators and these induce an increase in shell thickness in a number of species e.g. *Littorina* [3], *Argopecten* [59], *Mytilus edulis* [60], *Nucella* [56] and *Physella virgata virgata* [61]. In one instance, both chemical extracts of predators and damaged conspecifics were used and both produced an increase in shell thickness. The effect was additive, with the combined cues more effective than single applications [3]). Ice scour results in both mechanical disturbance and also chemical cues from extensively damaged benthic fauna and the resulting predator invasion [30]. In a temperate clam, with a similar morphology, *Mya arenaria*, chemical cues from green crabs have been shown to increase siphon length and burrowing depth [62]. There is no reason to discount this effect as the explanation for the data here. Given the variability in sediment depth in most of the Antarctic populations studied and the likely major effector, iceberg scour, deeper burying is probably not a common option and thicker shells are the more effective defence. Another surprising finding of this study is that individuals that had sustained and repaired even moderate or severe damage did not show a decrease in growth rate compared to undamaged or lightly damaged individuals. It is widely suggested that shell secretion is metabolically expensive [17] [18] and in the cold waters of Antarctica, where calcium carbonate is more soluble, one would expect this to be particularly so [63]. Certainly in the temperate example of *Mytilus edulis* in the Gulf of Maine, this energetic cost was demonstrated, as animals with thicker shells inhabited those environments with a higher food supply [64]. However, the Southern Ocean experiences a highly seasonal food supply and it may be that energetic trade-offs occur in damaged animals with, for example, potentially reduced reproductive effort or physiological capacities. Indeed, it has been found that there are physiological differences in *L. elliptica* between more and less disturbed sites [31]. More disturbed *L. elliptica* had different metabolic responses to increased sediment load in the water column. Interestingly, although it is a widely accepted paradigm that shell thickness declines with increasing latitude in molluscs [65] [66], and this was also recently demonstrated for echinoderms as well as molluscs, there are exceptions [67] In these species, ecological factors take precedence over this energetic constraint and one of these exceptions was the Antarctic clam *L. elliptica* which has a thicker shell than temperate and tropical congeners. In this study we further demonstrate the flexibility of this phenomenon within Antarctic waters in response to physical disturbance and damage. Given the future predictions of acidification of the oceans leading to surface waters of the Southern Ocean becoming under-saturated in calcium carbonate by the middle of this century [32], such flexibility and spare capacity could be essential for survival. Understanding the mechanisms, extent and control of such phenotypic plasticity will be crucial to predicting how this keystone species will fare under future environmental perturbation. In this respect, molecular studies are proving critical to understanding shell deposition in this non-model species [27].
